# Advances in Hemodynamic Analysis in Cardiovascular Diseases Investigation of Energetic Characteristics of Adult and Pediatric Sputnik Left Ventricular Assist Devices during Mock Circulation Support

**DOI:** 10.1155/2019/4593174

**Published:** 2019-11-15

**Authors:** Alexander A. Pugovkin, Aleksandr G. Markov, Sergey V. Selishchev, Leonie Korn, Marian Walter, Steffen Leonhardt, Leo A. Bockeria, Olga L. Bockeria, Dmitry V. Telyshev

**Affiliations:** ^1^Institute for Biomedical Systems, National Research University of Electronic Technology, Zelenograd 124498, Moscow, Russia; ^2^Institute for Bionic Technologies and Engineering, I. M. Sechenov First Moscow State Medical University, 119991 Moscow, Russia; ^3^Medical Information Technology, RWTH Aachen, 52074 Aachen, Germany; ^4^Bakulev Center for Cardiovascular Surgery, 121552 Moscow, Russia

## Abstract

The need to simulate the operating conditions of the human body is a key factor in every study and engineering process of a bioengineering device developed for implantation. In the present paper, we describe in detail the interaction between the left ventricle (LV) and our Sputnik left ventricular assist devices (LVADs). This research aims to evaluate the influence of different rotary blood pumps (RBPs) on the LV depending on the degree of heart failure (HF), in order to investigate energetic characteristics of the LV-LVAD interaction and to estimate main parameters of left ventricular unloading. We investigate energetic characteristics of adult Sputnik 1 and Sputnik 2 LVADs connected to a hybrid adult mock circulation (HAMC) and also for the Sputnik pediatric rotary blood pump (PRBP) connected to a pediatric mock circulation (PMC). A major improvement of the LV unloading is observed during all simulations for each particular heart failure state when connected to the LVAD, with sequential pump speed increased within 5000–10000 rpm for adult LVADs and 6000–13000 rpm for PRBP with 200 rpm step. Additionally, it was found that depending on the degree of heart failure, LVADs influence the LV in different ways and a significant support level cannot be achieved without the aortic valve closure. Furthermore, this study expands the information on LV-LVAD interaction, which leads to the optimization of the RBP speed rate control in clinics for adult and pediatric patients suffering from heart failure. Finally, we show that the implementation of control algorithms using the modulation of the RBP speed in order to open the aortic valve and unload the LV more efficiently is necessary and will be content of further research.

## 1. Introduction

Nowadays, about 8 million people in Russia suffer from heart failure (HF), and among them, approximately 2.5 million have acute HF (classes III and IV of the New York Heart Association (NYHA) classification of heart failure), which tends to be the most widespread cause for hospitalization and lethal outcome of heart diseases [1–3].

Left ventricular assist devices (LVADs) were designed as a therapeutic option to treat end stage HF patients in response to the large patient populations with acute HF along with limited number of donor hearts. Initially, LVADs were designed as pulsatile blood pumps to support or replace the native ventricle. Eventually, LVADs evolved to rotary blood pumps (RBPs) providing continuous flow to maintain temporary and permanent circulatory support [4, 5].

Implantation of a LVAD leads to a dynamic interaction between the cardiovascular system and the LVAD, where the LVAD is unloading the ventricle via undertaking part of the load and in some cases causing its recovery. In its turn, the ventricle function influences the performance of the LVAD [6]. In order to evaluate this interaction, in particular ventricular unloading and recovery, the following energetic characteristics are described in the literature: stroke work (SW), hydraulic pump work, and cardiac mechanical efficiency [7–9].

Investigation of in vitro dynamic of LVAD is of importance due to its influence on the LVAD patients' outcome. It has been shown that the introduction of energetic parameters could lead to improved cardiac mechanical efficiency [10]. Moreover, proper assessment of LVAD dynamic leads to decreased LV afterload and improved LV mechanical performance (i.e., increased ejection fraction, stroke volume, cardiac output, and maximum elastance) [11]. Finally, there have been made investigations of pulsatile operating conditions which showed that there is a variable phase relationship between LVAD differential pressure and LVAD flow, which is important for clinical use [12].

The clinical importance of pulsatility is a recurring topic of debate in mechanical circulatory support. Lack of pulsatility has been identified as a possible factor responsible for adverse events and has also demonstrated a role in myocardial perfusion and cardiac recovery. Moreover, additional benefits of pulsatile ventricular assist devices (VADs) over continuous-flow systems are the greater likelihood of aortic valve opening. Investigation on the dynamics and characteristics of the LVAD and the pulmonary artery pulsatile index is also of importance due to the possibility to predict the risk of the gastrointestinal bleeding in patients with LVAD [13]. This issue of gastrointestinal bleeding (GIB) occurs primarily from arteriovenous malformations or ulcers [14].

The interaction between the left ventricle (LV) and the RBP can be examined via pressure-volume (P-V) diagram of the ventricle as the stroke work can be expressed by the area inside P-V diagram. In addition, dynamic head pressure-bypass flow (H-Q) curves of the pump are a strong tool for the evaluation of the LV-LVAD interaction, since dynamic H-Q curves describe RPB behavior during the cardiac cycle [15]. Also, the stroke work of the ventricle expressed by the area inside the P-V diagram strongly correlates with the area inside dynamic H-Q curves [6].

In this study, the interaction between different Sputnik LVADs and the LV with various degrees of HF was investigated. The evaluated LVADs are the adult Sputnik 1 and Sputnik 2, and furthermore, the Sputnik pediatric RBP (PRBP) was engineered for pediatric patients with a body weight between 12 and 40 kg. This research is aimed (i) to evaluate the Sputnik LVADs and Sputnik PRBP influence on the LV depending on the HF degree, (ii) to estimate main parameters of the LV unloading, (iii) to investigate energetic characteristics of the LV-LVAD interaction for the adult Sputnik LVADs and for the Sputnik PRBP, and finally (iv) to compare this between two generations of adult LVADs. This research is intended to expand the information on LV-LVAD interaction in order to optimize the choice of RBP speed rate control in the clinic regarding the degree of heart failure in adult and pediatric patients.

## 2. Materials and Methods

In this work, energetic characteristics of (i) Sputnik 1 and (ii) Sputnik 2 connected to a hybrid adult mock circulation (HAMC) [5] and (iii) Sputnik PRBP connected to a pediatric mock circulation (PMC) [16] were investigated.

Herein, in this work, we describe two various mock loops with two different working fluids. The dynamic viscosity of the fluid in the HAMC is around 2.5 MPa s at 298.15 K, whereas the dynamic viscosity of the fluid in the PMC is 2.38 MPa s at 299.25 K. Both are within the physiological range of blood viscosity, so the results are comparable.

### 2.1. Sputnik LVAD of Generation 1

The Sputnik 1 LVAD configuration is based on an axial-flow blood pump with nonpulsatile flow. This device can provide a flow rate up to 10 l/min.

**Figure 1:**
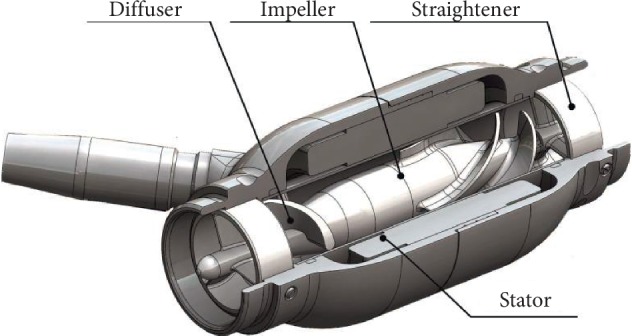
Profile of the Sputnik LVAD first-generation model. Four major parts of the device: (i) a straightener, (ii) a stator, (iii) an impeller, and (iv) a diffuser.

The profile of the blood pump is provided in Figure 1. The pump consists of two major parts: (i) a hydraulic and (ii) an electric. The hydraulic machine in its turn consists of an impeller (a rotor with 4 blades), a flow straightener with three blades, and a diffuser. An external power supply is used to drive the pump. This driving unit has two wearable batteries. The driver unit is connected to the pump by a percutaneous cable. An impeller with a permanent NdFeB magnet actuated by a brushless DC motor provides the blood flow through the Sputnik 1 LVAD. A stator is enclosed in a thin-walled titanium housing. The blood flow is directed by three inlet blades to the rotating impeller blades, minimizing the whirl flows. After this, blood flows into the diffuser. A diffuser consists of 3 twisted blades located at the pump outlet. These inlet and outlet elements have two different needle bearings: the inlet and the outlet. An impeller is suspended between these bearings.

The length of Sputnik 1 LVAD pump is 82 mm, the maximum pump diameter is 34 mm, the flow channel diameter is 16 mm, the impeller diameter is 15.6 mm, and finally the weight of the construction is 246 g [17].

### 2.2. Sputnik LVAD of Generation 2

Eventually, the Sputnik 1 LVAD was modified into Sputnik 2 LVAD. The bearing mountings of the flow straightener and diffuser were reduced significantly. The design of the diffuser was changed in order to exclude the rotor taper unit expansion and also to reduce the weight and the size of the pump. The distance between the impeller and the diffuser was changed from 20 mm to 3.6 mm. The pressure head, as a result, was increased with the decrease in distance [18]. The overall changes bring the possibility to decrease the pump's length. The rotor was tightened by 2 repelling magnets in the outlet bearing. Therefore, during on and off states of the device, a permanent surface contact is formed. Schematic and geometry of the diffuser of the first and second generations of the Sputnik LVADs are demonstrated in the work of Selishchev and Telyshev [19].

The implantable pump length was decreased from 81 mm (the HMII and HA5 lengths are 81 and 71 mm, respectively) to 66 mm. The maximum diameter was decreased from 34 mm (the HMII and HA5 maximum diameters are 43 and 30 mm, respectively) to 29 mm [20]. The diameter of the impeller was decreased from 15.6 mm to 13.8 mm, and the pump weight from 246 g to 205 g. Finally, a new geometry of the diffuser and the modification of the rotor design allowed to reduce the energy consumption of the device.

### 2.3. Sputnik PRBP

The necessity to develop a device for pediatric patients imposed many obstacles. Therefore, the first phase of the Sputnik PRBP design involved the detailed elaboration of requirements for the pediatric patients, which significantly differed from the adult group [21]. The two main differences of the cardiovascular system in pediatric patients compared to adults are (i) a higher heart rate and (ii) lower arterial blood pressure. Another key factor is the cardiac output, which is noticeably increased in adult patients [22].

**Figure 2:**
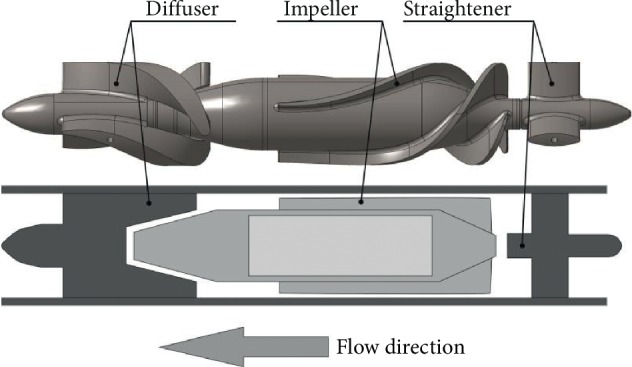
Schematic (bottom) and geometry (top) of the hydraulic part of the Sputnik PRBP.

The working principle and design features of Sputnik PRBP are described in detail in the work of Dr. Telyshev et al [23]. Herein, we follow the methods of Dr. Telyshev to list these features. A construction scheme that was successfully used for development of the two adult Sputnik LVADs was utilized as a basis for the Sputnik PRBP development. As shown in Figure 2, hydraulic part of the Sputnik PRBP consists of a fixed flow tube containing the main components of the pump: (i) a fixed flow straightener at the input, (ii) an impeller with an embedded magnet allocating a rotational speed of several thousands of revolution per minute (rpm), and (iii) a fixed diffuser at the output. The flow straightener has three blades arranged at an angle of 120° to each other. To reduce the residence time of blood cells in the pump, the distance between the back edge of the impeller blades and the front edge of the diffuser blades was decreased from 20 mm (Sputnik 1 LVAD) to 6.5 mm. It is presumed that with a reduction of the residence time of blood in the pump, the risk of blood trauma decreases and, therefore, the pump's biocompatibility increases. The lowest hemolysis index was observed when the distance between the impeller and diffuser was 2 mm or 6 mm. A flow rate of 2.4 l/min is defined as an operating point of the Sputnik PRBP. The use of the adult Sputnik LVAD at this operating point unavoidably leads to decreased velocity of blood cells in the pump [18] and hence to an increased residence time, providing increased probability of blood cell trauma. The Sputnik PRBP has the following geometrical specifications: flow unit length of 51.5 mm, flow unit diameter of 10 mm, and spacing between the rotor blades and housing of 0.1 mm.

The main parts of the pump are made of titanium alloy, whereas the bearings used in the pump are made of CoMoCr alloy, as in the adult Sputnik LVADs. Moreover, the rotor of the Sputnik PRBP contains a permanent NdFeB magnet actuated by a brushless DC motor, the same as used in the first and second generations of the adult Sputnik LVADs.

Thus, Sputnik PRBP is suitable for pediatric patients with body weight of 12–40 kg.

### 2.4. Hybrid Adult Mock Circulation

**Figure 3:**
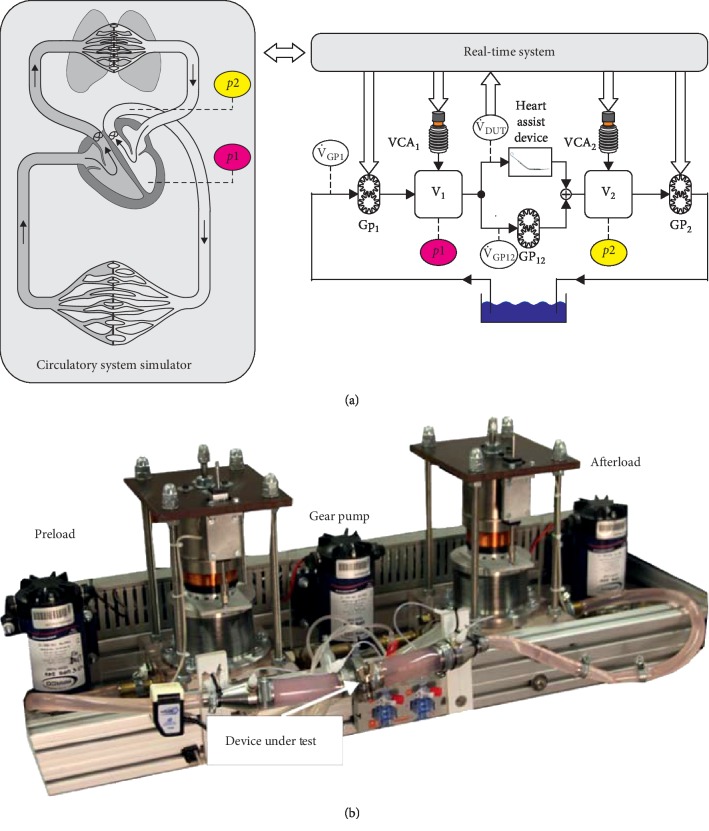
(a) Hydrodynamic system simulator (right) coupled to the circulatory system simulation (left). The left ventricular pressure p1 (preload) and the aortic arch pressure p2 (afterload) are calculated via the simulation and used as reference values for the hybrid adult mock circulation. Measured values in the experimental setup are indicated by ellipsoids, modified from the literature values. (b) Photo of the hybrid adult mock circulation (HAMC). The preload and afterload pressure compartments with the attached voice coil actuators can be seen as well as the three gear pumps providing the flow balance in the system. The device under test is mounted in the fixation in front. A detailed description of the system can be found in [3].

For the study of the adult Sputnik LVADs, a mock circulation loop [5] based on a numerical cardiovascular model for adults was used [24]. In the experiment, the device is connected to a real-time computer (DS1103, dSPACE GmbH, Paderborn, Germany) via a hydrodynamic interface using the Hardware in the Loop (HIL) concept. High-bandwidth actuators are used to control the left ventricular and the aortic pressure in the test bench while the pump flow is fed back into the numerical simulation (Figure 3).

In comparison to classical mock circulation loops, herein the heart and the cardiovascular system are exclusively modeled in the numerical domain and transferred into the physical domain as set points for the pump inlet and outlet. The cardiovascular system simulation is implemented as a simplified version of the model from [24] in the software Simulink. Eight ordinary differential equations and eight state variables are used to model an actively contracting LV, including both the atrium and the ventricle. The control input is set to appear between a certain maximum and minimum flow level. Frank–Starling autoregulation mechanism of the heart is reproduced during the simulation. Physiological control loops like the baroreceptor reflex are also modeled. The control of the cardiovascular system is responsible for the adaptation of the heart rate and the contractility of the ventricle. The transition between the end-diastolic and the end-systolic pressure to the volume (*P*_ed_(*V*),  *P*_es_(*V*)) is modeled by chamber-specific time-varying elasticity functions *e*(*t*), that result in the function of pressure *p*(*V*, *t*) for each chamber.

(1) $$\begin{array}{c} p\left(V,t\right)=e\left(t\right)\cdot P_{\text{es}}\left(V\right)+\left(1-e\left(t\right)\right)\cdot P_{\text{ed}}\left(V\right);\\ P_{\text{es}}\left(V\right)=\text{VCF}\cdot E_{\text{es}}\cdot \left(V\left(t\right)-V_{\text{u}}\right), \end{array}$$

where *E*_es_ describes the chamber-specific elasticity and can be modified by the nondimensional contractility factor 0 < VCF ≤ 1 and *V*_u_ describes the volume when the transmural pressure is equal to zero and can be set individually for each chamber.

The hardware part of the mock circulation loop contains mainly two compartments made of polymethyl methacrylate and does not contain any valves, compliance chambers, or flow resistances. The chambers are actuated via voice coil actuators (VCAs) to reproduce fast pressure changes, whereas three gear pumps generate directed flow to keep the VCAs in the operating range. The adult mock circulation loop is capable to achieve an accuracy of ±1 mmHg and a settling time less than 20 ms. Invasive blood pressure sensors (Xtrans, CODAN pvb Critical Care GmbH, Forstinning, Germany) are used to measure pressures in two compartments, whereas an ultrasonic flow probe is utilized to determine the pump flow (H11XL, Transonic Systems Inc., Ithaca, USA). In addition, the hydrodynamic properties of blood are emulated with a glycol/water mixture (Glysofor N, Wittig Umweltchemie GmbH, Grafschaft-Ringen).

### 2.5. Pediatric Mock Circulation

**Figure 4:**
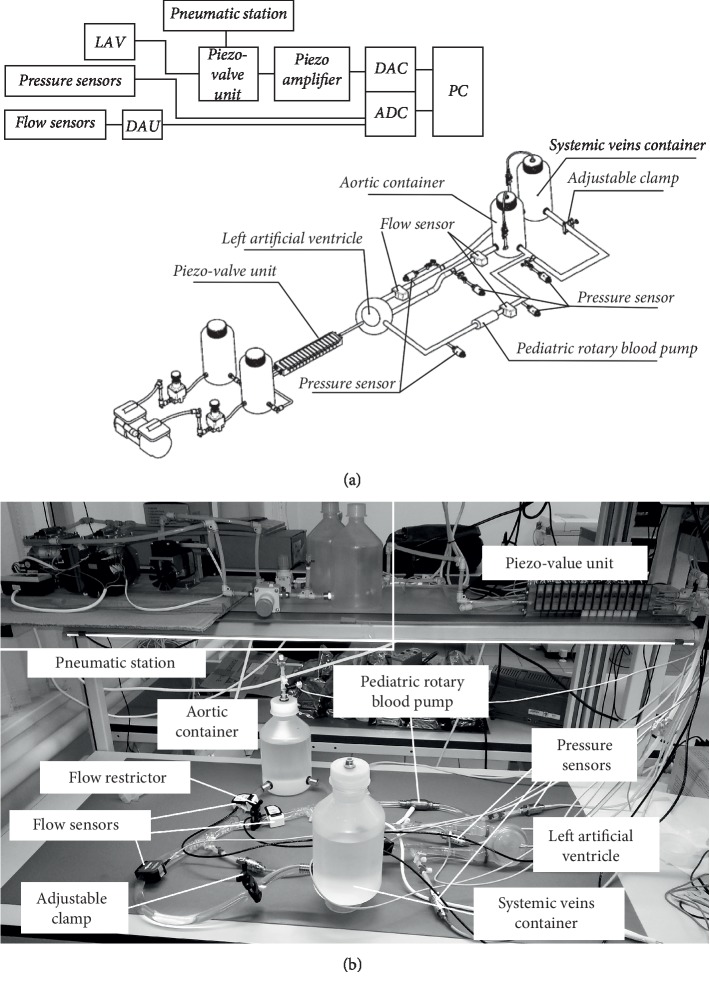
(a) Schematics of PMC showing main functional hardware blocks. LAV, left artificial ventricle; DAU, data acquisition unit; ADC, analog-to-digital converter; DAC, digital-to-analog converter; PC, personal computer. (b) Image of the pediatric mock circulation (PMC) simulating the acute heart failure with the Sputnik PRBP support. The flow direction is shown via an arrow.

For the test under dynamic conditions with the Sputnik PRBP, a conventional pediatric mock circulation (see Figure 4) allowing for the simulation of physiological cardiovascular characteristics was utilized [16]. The simulation was automatized using NI cDAQ hardware and NI LabVIEW software. This system being a physical-based model of pediatric circulation reproduces the Frank–Starling autoregulation mechanism of the heart, which regulates the cardiac output depending on the ventricle preload. This can be expressed as end-diastolic pressure (EDP) or end-diastolic volume (EDV). The numerical model of the cardiovascular system was not used during trials of the Sputnik PRBP considering its inconsistence for pediatric conditions by the moment of trials.

The system in Figure 4 allows to simulate and control the cardiovascular circulation and consists of a pediatric systemic circulation loop (PSCL), a pneumatic system, and the data acquisition system. The PSCL consists of an artificial ventricle (AV) (Medos LVAD; Medos Medizintechnik AG, Stolberg, Germany), two containers reproducing lumped vascular parameters, and an adjustable clamp to reproduce the systemic vascular resistance. All components of the PSCL are connected in series with flexible polyvinyl chloride laboratory tubes (TYGON E-3603; Compagnie de Saint-Gobain, Courbevoie, Ile-de-France, France), with an inner diameter of 12.7 mm.

The left pulsatile AV has a nominal volume of 72 ml corresponding to a dilated pediatric ventricle volume. It is a pump pneumatically driven by a membrane that simulates the heartbeat using the pneumatic control system. The pulsatile AV has an inlet and an outlet valve to prevent reverse flow of fluid into the hydraulic circuit. A 32% aqueous glycerol solution is used in the PSCL as the model fluid. This configuration of the mock circulation allows simulating normal pediatric systemic circulation and heart failure state. It should be clarified here that under the normal state, we mean summation of LV failure state with connected LVAD, and this applies to both flow and work. The left AV also has a second outlet providing the connection between the Sputnik PRBP inlet and the ventricle. The Sputnik PRBP outlet is connected to the aortic container. Before testing the Sputnik PRBP behavior and its influence on the system, the pump connection line was closed with a special flow restrictor.

The pneumatic system including a pneumatic station and a pneumatic control unit is used to control the AV contraction through an electronic control system based on combined NI cDAQ-9174 and NI 9264 hardware implemented with the NI LabVIEW software (National Instruments Corporation, Austin, Texas, USA). The contractility level of the pulsatile AV can be varied by altering the control signal amplitude and frequency. The Frank–Starling mechanism is reproduced by using EDP as a preload parameter in feedback.

The data acquisition system consists of a pressure measurement system, a flow measurement system, and a data processing system based on combined NI cDAQ-9174 and NI 9205 hardware and NI LabVIEW software.

### 2.6. Characteristics Analysis

Adult LVADs characteristics were analyzed in two operation modes of the HAMC. Each operation mode is characterized by nondimensional ventricle contractility factors (VCFs) of 0.5 and 0.25. These two operation modes correspond to mild and congestive heart failure states, respectively. An acute heart failure state in pediatric patient with the body mass of 15.2 kg [25] was simulated on PMC to investigate the energetic characteristics of the Sputnik PRBP.

The energetic characteristics of Sputnik 1 and Sputnik 2 LVADs were obtained in a speed range of 5000–10000 rpm increasing in steps of 200 rpm. Sputnik PRBP operation was analyzed in the speed range of 6000–13000 rpm in steps of 200 rpm.

The left ventricular stroke work, LVAD hydraulic work, and total work investigated from the energetic characteristics can be described as follows:

(2) $$\begin{array}{c} A_{\text{LV}}=\oint P_{\text{LV}}\text{d}V_{\text{LV}}, \end{array}$$

where *P*_LV_ is the left ventricular pressure and *V*_LV_ is the left ventricular volume.

(3) $$\begin{array}{c} A_{\text{VAD}}=\int \left(P_{\text{out}}-P_{\text{in}}\right)Q_{\text{VAD}}\text{d}t, \end{array}$$

where *P*_out_is the LVAD outlet pressure, *P*_in_is the LVAD inlet pressure, and *Q*_VAD_is the LVAD flow rate.

(4) $$\begin{array}{c} A_{\text{Total}}=A_{\text{LV}}+A_{\text{pump}}. \end{array}$$

Left ventricular stroke work represents the area inside the closed contour formed by the left ventricular pressure-volume relationship. The LVAD hydraulic work, in its turn, is the time integral of the LVAD head pressure and flow rate product. Total work is the sum of the *A*_LV_ and *A*_VAD_.

Also, the mean LVAD flow rate, the mean aortic valve flow rate, the end-diastolic volume, and the relationship between left ventricular stroke work and the LVAD hydraulic work were utilized to describe all simulated conditions. The characteristics were calculated for each cardiac cycle on a sample of obtained experiment data and were averaged over all cardiac cycles in each simulated condition.

Furthermore, pulsatility indices of pump flow rates were analyzed:

(5) $$\begin{array}{c} PI_{Q_{\text{pump}}}=\frac{Q_{\text{pump}}^{\max}-Q_{\text{pump}}^{\min}}{Q_{\text{pump}}^{\text{av}}}, \end{array}$$

where *Q*_pump_^max^, *Q*_pump_^min^, and *Q*_pump_^av^ are maximum, minimum, and average pump flow rates.

## 3. Results

**Figure 5:**
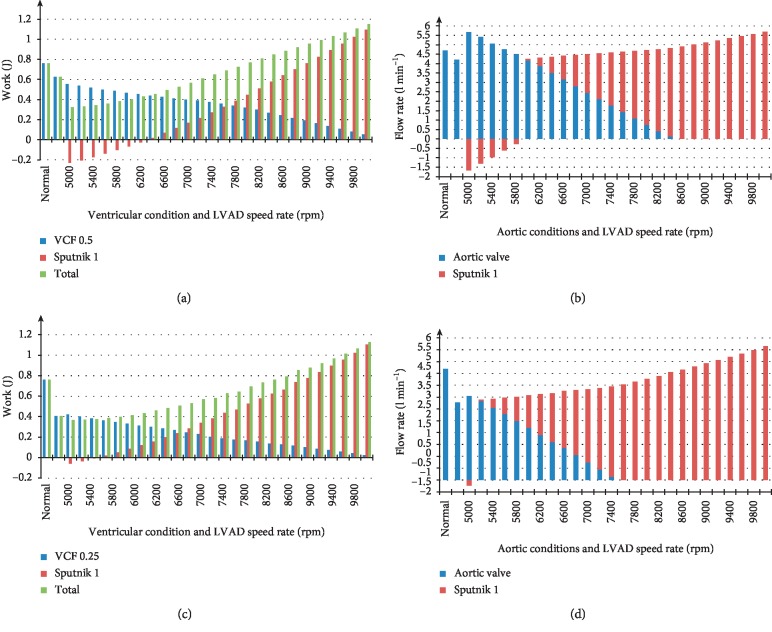
(a, c) Average levels of left ventricular stroke work, hydraulic pump work, and total work for Sputnik 1 LVAD supporting the adult LV with ventricular contractility factors (VCFs) of 0.5 and 0.25, respectively, in the pump speed range of 5000–10000 rpm with 200 rpm step. (b, d) Average aortic valve and pump flow rates with respect to normal, heart failure, and supported states in the pump speed ranges specified for Sputnik 1 LVAD supporting LV with VCFs of 0.5 and 0.25, respectively. Stroke work and aortic valve flow rates in normal and heart failure states are presented for comparison.

Figures 5(a) and 5(c) represent average levels of LV stroke work, the hydraulic work of the Sputnik 1 LVAD, and the total work during support of the LV with VCFs of 0.5 and 0.25, respectively. Figures 5(b) and 5(d) show bar graphs of the average aortic valve and pump flow rates for the operation modes of the LV with VCFs of 0.5 and 0.25, respectively, in the pump speed range of 5000–10000 rpm increasing in steps of 200 rpm. Normal and heart failure state levels of stroke work and the aortic valve flow rate are also presented for comparison at the left side of each figure.

The work of the Sputnik 1 pump intersects with zero in the speed range of 6200–6400 rpm for a VCF of 0.5 and at 5400–5600 rpm for a VCF of 0.25 (Figures 5(a) and 5(c)). The total work reaches a value of the normal LV work equal to 0.76 J at 8000 rpm and 8400 rpm for VCFs of 0.5 and 0.25, respectively.

Figures 5(b) and 5(d) show that the total average flow rates for VCFs of 0.5 and 0.25 are equal to the normal LV flow rate in the speed ranges of 7800–8000 rpm and 8600–8800 rpm, respectively. While the ventricle is still actively pumping, partial support takes place as it changes to full support when the aortic valve closes and the ventricle stops pumping. This happens at 8600 rpm and 7600 rpm for VCFs of 0.5 and 0.25, respectively.

**Figure 6:**
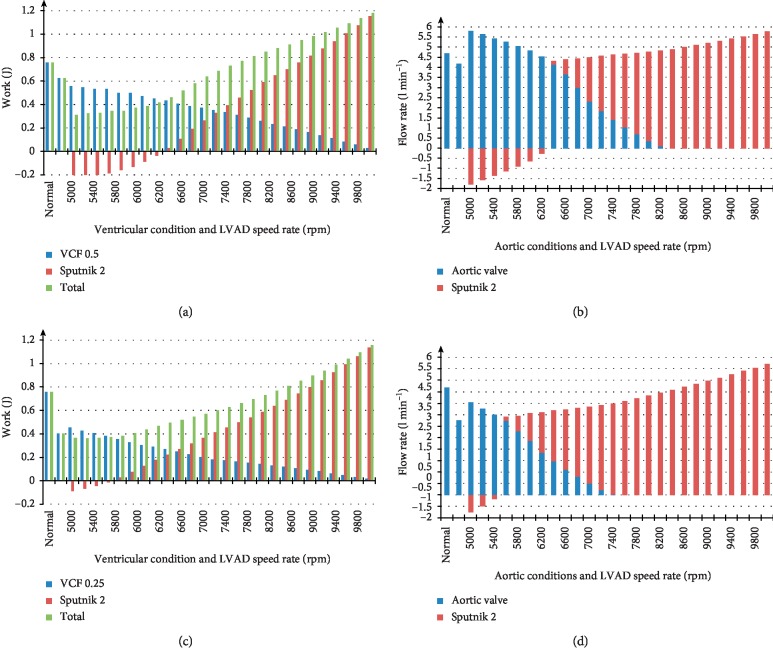
(a, c) Average levels of left ventricular work, hydraulic pump work, and total work during Sputnik 2 LVAD support with ventricle contractility factors (VCFs) of 0.5 and 0.25, respectively, in the pump speed range of 5000–10000 rpm with 200 rpm step. (b, d) Average aortic valve and pump flow rates with respect to normal, heart failure, and supported states in the pump speed ranges specified for Sputnik 2 LVAD supporting LV with VCFs of 0.5 and 0.25, respectively. Stroke work and aortic valve flow rates in normal and heart failure states are presented for comparison.

Figures 6(a) and 6(c) represent the average levels of left ventricular work, the hydraulic work of the Sputnik 2 LVAD, and the total work during support of the LV with VCFs of 0.5 and 0.25, respectively. Figures 6(b) and 6(d) show bar graphs of the average aortic valve and pump flow rates for LV operation modes with VCFs of 0.5 and 0.25 with respect to each figure. The pump speed range has been set to 5000–10000 rpm increased by 200 rpm per step.

Sputnik 2 pump work intersects with zero in the speed ranges of 6400–6600 rpm and 5600–5800 rpm for VCFs of 0.5 and 0.25, respectively (Figures 6(a) and 6(c)). The total work equals to the LV work in normal state at 7800 rpm and 8400 rpm for a VCF of 0.5 and 0.25, respectively.

Figures 6(b) and 6(d) show that the total average flow rates equal to the normal LV flow rate in the speed ranges of 7600–7800 rpm and 8400–8600 rpm, respectively. The partial support state of the Sputnik 2 LVAD changes to full support state at 8400 and 7400 rpm for VCFs of 0.5 and 0.25, respectively.

We chose one of the Medos VAD ventricles the aortic valve leaflets of which are prolapsed as an overload on the aortic valve increase. Thus, aortic back flow is reached after a certain pump speed.

**Figure 7:**
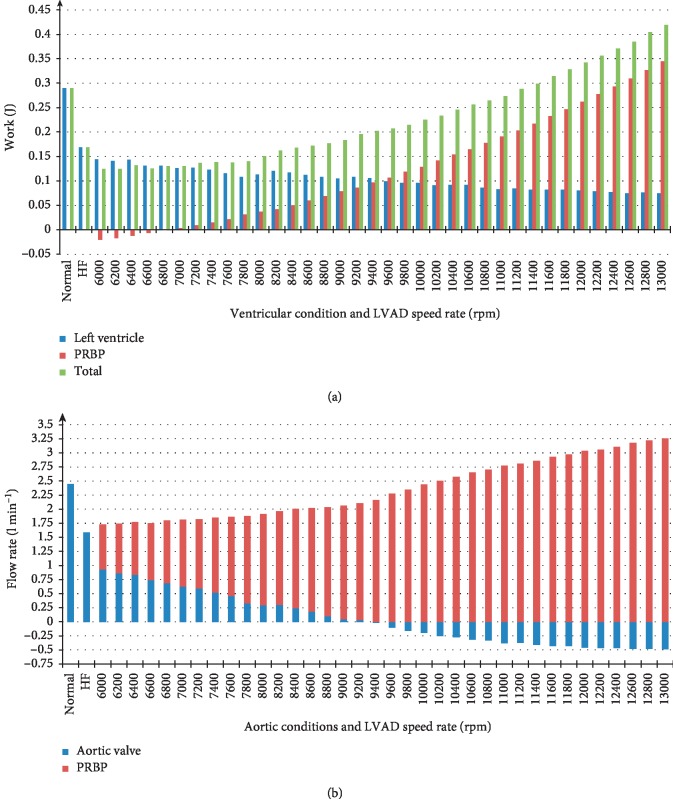
(a) Average levels of left ventricle work, PRBP work, and total work for simulated state of acute heart failure in 2-year-old pediatric patient with mass of 15.2 kg in the pump speed range of 6000–13000 rpm with 200 rpm step. Levels of left ventricle work in normal and heart failure states are also represented for comparison. (b) Average aortic valve and pump flow rates with respect to normal state, heart failure, and states in the pump speed range specified.

Figure 7 represents the average levels of LV work, the hydraulic work of the Sputnik PRBP, and the total work. In addition, a bar graph of the average aortic valve and pump flow rates simulated in the PMC with an acute heart failure state in the pediatric patient with a body mass of 15.2 kg is shown in the pump speed range of 6000–13000 rpm with 200 rpm step.

Figure 7(a) shows that Sputnik PRBP hydraulic work intersects with zero in a pump speed range of 6800–7000 rpm. The total work reaches the normal LV work at 11200 rpm.  Figure 7(b) shows that the total average flow rate equals to the normal LV flow rate at a pump speed of 11400 rpm. Sputnik PRBP partial support state changes to full support at a speed of 9400 rpm. The aortic back flow appears at a speed of 9400 rpm and the maximum of aortic back flow is reached at 13000 rpm.

**Figure 8:**
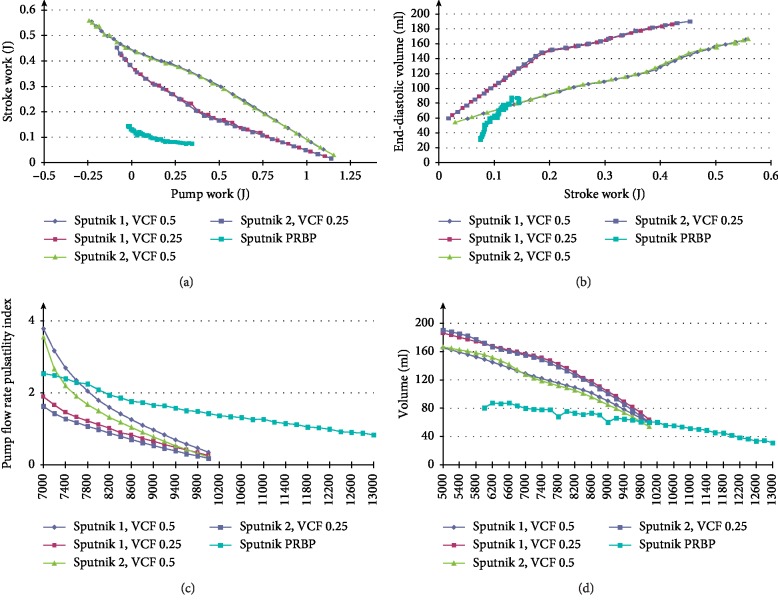
(a) Relationship between average stroke work and average hydraulic pump work for Sputnik 1 and Sputnik 2 LVADs supporting left ventricle with ventricle contractility factors (VCFs) of 0.5 and 0.25 and for Sputnik PRBP supporting pediatric left ventricle. (b) Relationships between end-diastolic volume and average stroke work for adult and pediatric LV supported by Sputnik 1, Sputnik 2 LVADs, and Sputnik PRBP, respectively. (c) Pump flow rate pulsatility indices for Sputnik 1 and Sputnik 2 LVADs supporting left ventricle with ventricle contractility factors (VCFs) of 0.5 and 0.25 and Sputnik PRBP supporting pediatric left ventricle in the speed ranges of 7000–10000 rpm and 7000–13000 rpm, respectively, with 200 rpm step. (d) End-diastolic volumes of left ventricle during support by Sputnik 1 and Sputnik 2 LVADs and Sputnik PRBP in the speed ranges of 5000–10000 rpm and 6000–13000 rpm, respectively, with 200 rpm step.

Figure 8(a) represents the relationship between average stroke and pump work for Sputnik 1 and Sputnik 2 LVADs supporting the LV with VCFs of 0.5 and 0.25 and the Sputnik PRBP supporting the pediatric LV in the acute heart failure state.  Figure 8(b) shows the relationship between the end-diastolic volume (EDV) and the average stroke work for adult and pediatric LVs during support.  Figure 8(c) represents pump flow rate pulsatility indices for Sputnik 1 and Sputnik 2 LVADs supporting the adult LV with VCFs of 0.5 and 0.25 in the speed range of 7000–10000 rpm in steps of 200 rpm. Furthermore, the Sputnik PRBP support in failing pediatric LV in the speed range of 7000–13000 rpm with 200 rpm steps is depicted. Also, Figure 8(d) shows EDV of the left ventricle during support by Sputnik 1 and Sputnik 2 LVADs in a speed range of 5000–10000 rpm (steps of 200 rpm) and Sputnik PRBP in the speed range of 6000–13000 rpm (steps of 200 rpm), respectively.

Relations represented in Figure 8 are almost the same for Sputnik 1 and Sputnik 2 LVADs in corresponding ventricular states with VCFs of 0.5 and 0.25. Figure 8(a) shows that the stroke work may depend on pump work as they are inversely proportional.  Figure 8(b) shows that EDV increases with stroke work. All pump flow rate pulsatility indices and the EDV decrease with pump speed for all presented RBPs.

**Figure 9:**
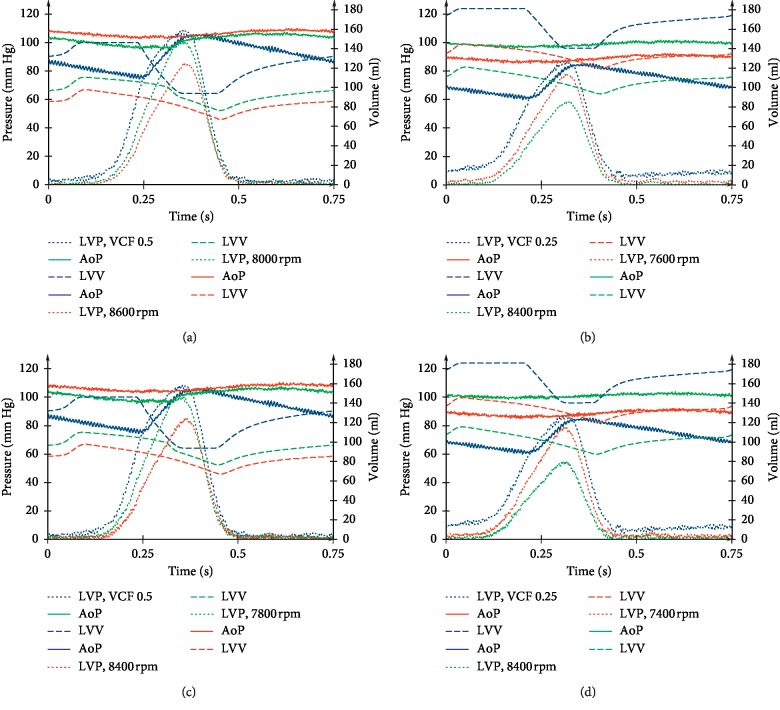
Time courses of the left ventricular (LV) pressure, the volume, and the aortic pressure for moderate and congestive heart failure states simulated with the HAMC at the pump speeds of aortic valve closure (red) and total work attainment of a normal LV stroke work level (green): (a) and (b) during Sputnik 1 LVAD support; (c) and (d) during Sputnik 2 LVAD support. Blue lines represent time courses of condition characteristics simulated without support. Dot lines demonstrate the left ventricle pressure, single lines demonstrate the aortic pressure, and dash lines represent the left ventricle volume.

Figure 9 represents time courses of the LV pressure and volume and the aortic pressure for moderate and congestive heart failure states simulated in the HAMC with and without the support of the adult Sputnik LVADs. It is shown that for Sputnik 1 (Figures 9(a) and 9(b) and Sputnik 2 (Figures 9(c) and 9(d)), as the rotor speed increases, the systolic pressure in the ventricle decreases and the volume curve moves towards decreasing end-systolic and end-diastolic volume. In addition, the aortic pressure increases, but the pulse pressure decreases with increasing pump speed due to its constancy. These changes indicate the unloading of the ventricle and the improvement of hemodynamics due to an increase in the average pressure in the aorta.

**Figure 10:**
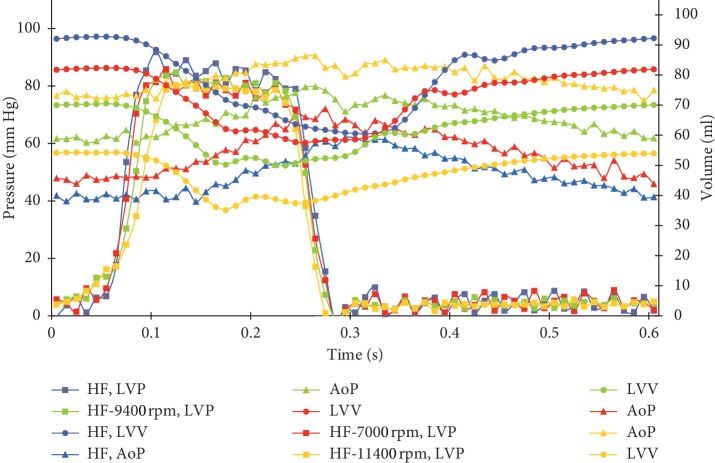
Time courses of the left ventricular (LV) pressure and volume; aortic pressure for acute heart failure in pediatric patient with body weight of 15.2 kg at the various pump speeds for partial support (red), partial-to-full support change (green), and total work attainment of a normal LV stroke work level (yellow). Blue lines represent time courses of condition characteristics simulated without support.

Figure 10 represents the time courses of the LV pressure and volume and the aortic pressure for acute HF in pediatric patients with and without the support of the Sputnik PRBP. Overall, the reproduced state of the pediatric cardiovascular system is very similar to the state of an adult blood circulation during heart failure. The comparison of partial and full support with the condition without support of the Sputnik PRBP shows that the systolic pressure in the ventricle decreases, the ventricular volume decreases, and the pressure in the aorta increases, which leads to unloading of the ventricle and an improvement of general hemodynamics.

**Figure 11:**
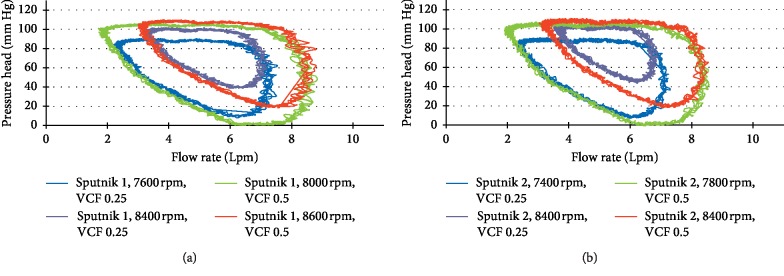
Dynamic pressure head-flow rate curves of Sputnik 1 and Sputnik 2 LVADs supporting LV in the moderate and the congestive heart failure state at the pump speeds of partial-to-full support change (blue and red) and full support state (green and purple).

**Figure 12:**
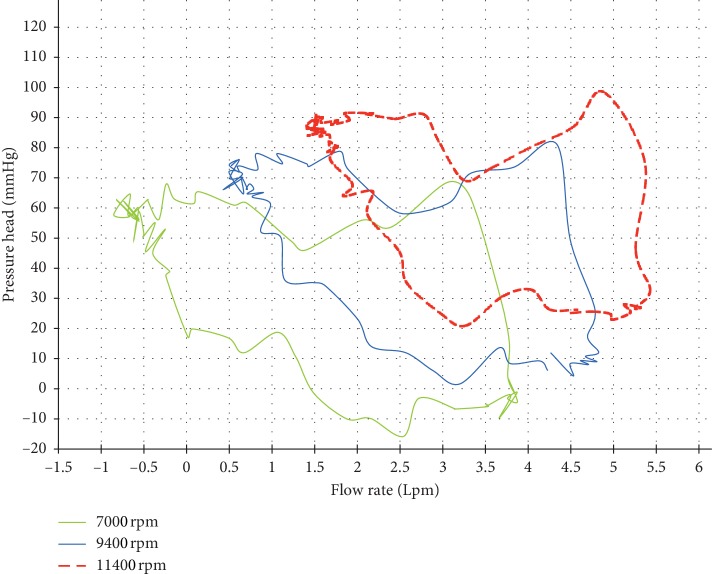
Dynamic pressure head-flow rate curves of Sputnik PRBP supporting failing pediatric LV at the pump speeds for the partial support state (green), the partial-to-full support change (blue), and the full support state (red) with the total work attaining normal LV stroke work level.

Figure 11 represents dynamic H-Q curves of Sputnik 1 (Figure 11(a)) and Sputnik 2 (Figure 11(b)) LVADs supporting moderate and congestive HF states of the LV at pump speeds of partial-to-full support change and total work attainment of the normal LV stroke work level.  Figure 12 represents dynamic H-Q curves of the Sputnik PRBP in corresponding states. In Figure 11, it is shown that, during the filling of the ventricle, the pressure in the pump remains constant (top flat area of the figure) with changing flow in the pump, whereas its value approximately corresponds to the average aortic pressure of about 100 mmHg. A similar observation can be made in Figure 12. The flow rate and the pressure increases with higher rpm. However, in this case, the mean aortic value corresponds to the average value of the pressure head during the ventricular filling phase. The pressure during the filling phase becomes less stable in comparison to the adult models (Figure 11), due to limitations imposed by the miniaturization of the device for pediatric patients.

## 4. Discussion

The unloading tendency of the LV is observed in all simulations for all heart failure states with a connected LVAD, when the pump speed increases within a certain range. This, coupled with an increased average hydraulic pump work, leads to a systemic circulation performance improvement. This is also clearly indicated by the development of the average total work and the average total flow rate of the LV and the LVAD. The change from the partial support to full support is accompanied by the aortic valve closure followed by an aortic back flow. This behavior occurs at a certain operation mode for each LVAD and depends on the residual LV contractility. Thus, based on this operation mode, the LVAD becomes the only fluid actuator in the circulatory system.

Figures 4–6 show that characteristics of the total average work of the LV and LVAD linearly increase with pump speed. This happens also in the speed ranges of 5000–6200 rpm and 5000–5400 rpm for Sputnik 1 LVAD at VCFs of 0.5 and 0.25, respectively, and 5000–6400 rpm and 5000–5600 rpm for Sputnik 2 LVAD at VCFs of 0.5 and 0.25, respectively. If the average hydraulic pump work is negative, the pumps do not provide any significant support for the LV. This is observed in the pump flow rates within specified speed ranges, which are negative as well. Further pump speed increase causes the hydraulic pump work to become higher. Moreover, the total work of the LV and LVAD grows leading to more significant support and unloading of the LV.

In the case of the Sputnik PRBP, an average hydraulic pump work is negative in the speed range of 6000–7800 rpm, although the flow rates are positive. Also, there is an obvious correlation between the average aortic valve flow rate and the average hydraulic pump work, i.e., the hydraulic pump work exceeds the LV stroke work right after the aortic valve closure. This correlation does not match the adult Sputnik LVADs tendency, as the hydraulic pump work overshoots the LV stroke work before the aortic valve closes.

The dependencies between the EDV and the stroke work for the Sputnik 1 and Sputnik 2 LVADs at a VCF of 0.25 have an inflection point, which corresponds to an aortic valve closure. At this point, the slope of the EDV becomes steeper as the stroke work decreases.

Due to improvements in the design, the Sputnik 2 LVAD allows to achieve LV normal state characteristics slightly quicker than Sputnik 1 LVAD, i.e., at lower pump speed. An exception needs to be done for the LV and LVAD total work at a VCF of 0.25 which is achieved at the same speed as the Sputnik 1 LVAD. Almost simultaneously, the normal state work and the flow rate are achieved by the Sputnik 1 and Sputnik 2 LVADs, i.e., at similar pump speeds. In the case of the Sputnik PRBP, a normal level of the total work is achieved at a slightly lower speed in comparison to the normal flow rate. However, the speed difference is only 200 rpm. Thus, obtaining the normal average flow rate during a support with the Sputnik PRBP, it can be seen that the total work of a normal state along with sufficient LV unloading is achieved.

EDV is another indicator of the LV unloading, since it decreases with increasing pump speed (Figure 8(d)). The correlation between EDV and the pump speed for VCFs of 0.5 and 0.25 is almost equal for Sputnik 1 and Sputnik 2 LVADs. Also, the relationship between the average stroke work and the average hydraulic pump work is very similar for Sputnik 1 and Sputnik 2 LVADs at corresponding VCFs. It can be concluded that the results of the experiments for Sputnik 1 and Sputnik 2 LVADs show very strong interrelations in spite of constructive differences.

All pump flow rate pulsatility indices of the adult Sputnik LVADs for different VCFs converge to zero at high pump speeds (over 9000 rpm). This behavior is most likely brought by the fact that the LV, as the only pulsatile element of the entire system, ceases to pulse being unloaded with an increase in pump speed. Therefore, all characteristics including the pump flow rate become less pulsatile. The flow rate pulsatility index of the Sputnik PRBP shows the same but more gradual trend with respect to pump speed.

The presented research shows that RBPs differently influence the LV depending on heart failure degree. Thus, significant support levels cannot be obtained for a VCF of 0.25 in the HAMC and for pediatric LV in the PMC without an aortic valve closure in comparison to a VCF of 0.5 in the HAMC. To solve this problem, the implementation of control algorithms providing a modulation of the LVAD speed in order to open the aortic valve and to unload the LV more efficiently is needed. Moreover, the importance of maintaining pulsatility has been suggested in many journals. There are restrictions for mechanical support when a patient's health is in a very poor condition. Algorithms of the physiological control may be exceptionally useful to overcome this issue. Ando et al. has recently created a pulsatile mode for continuous-flow left ventricular assist devices which can produce pulsatility comparable to the physiological pulsatile flow [26]. Bozkurt et al. showed that it is possible to obtain more physiological pulsatile hemodynamics in the arteries by applying output-driven varying speed control to a CF-LVAD [27]. Soucy et al. conducted a research where it was shown that pump speed modulation increases pulsatility and improves cardiac function and endorgan perfusion [28]. The same applies to investigation conducted regarding pulsation generation [29]. For instance, Vincent et al. showed that the modulation of von Willebrand factor levels could explain the relationship between pulsatility and bleeding observed in CF-MCS recipients [30]. Whereas, Edwards et al. showed that low pulsatility index is associated with an increased hazard of overt gastrointestinal bleeding in their cohort of HeartMate II recipients [31].

### 4.1. Limitations

An absence of the aortic back flow in the HAMC should be considered as a limitation of the presented study, since this condition is frequently observed in patients during LVAD support on relatively high speeds and its presence could lead to more accurate results for the adult Sputnik LVADs.

The Frank–Starling mechanism is simulated according to equations for ventricular pressure and volume. It should be noted that there is no automated feedback mechanism simulating Frank–Starling mechanism in the pediatric mock circulatory system in this study. However, in our case, the Frank–Starling mechanism is implemented according to previously manually selected parameters of contractility and finite diastolic volume.

## 5. Conclusion

The present work underlines the importance to simulate operating conditions of the human body during the development of a bioengineering device designed for implantation. The significance of pulsatility is highlighted in this work in addition to its importance in clinical applications (i.e., the cardiac recovery in patients and possibility to predict the risk of the gastrointestinal bleeding in patients with LVAD). In this work, we investigated the interaction between different Sputnik LVADs and the LV with different degrees of HF. We evaluated various LVADs: adult Sputnik 1, Sputnik 2, and Sputnik pediatric RBP (PRBP) engineered for pediatric patients with body a weight between 12 kg and 40 kg. We show the influence of the Sputnik LVADs on the LV depending on the HF degree by the estimation crucial parameters of LV unloading and investigation energetic characteristics of the LV-LVAD interaction for adult and pediatric Sputnik LVADs. Finally, we state that since significant support levels cannot be obtained for a VCF of 0.25 in the HAMC and for pediatric LV in the PMC without the aortic valve closure in comparison to a VCF of 0.5 in the HAMC, it is necessary to investigate control algorithms providing a modulation of the LVAD speed in order to open the aortic valve and unload the LV more efficiently.

## Data Availability

All the data used to support the findings of this study are available from the corresponding author upon request.
